# Associations of antimicrobial and multidrug resistance in *Escherichia coli* from the fecal flora and housing environment of calves on dairy farms with atmospheric variables

**DOI:** 10.1128/msphere.00847-25

**Published:** 2026-04-30

**Authors:** Véronique Bernier Gosselin, Martin Wegmann, Romane Zufferey, Vincent Perreten, Mireille Meylan

**Affiliations:** 1 Clinic for Ruminants, Vetsuisse Faculty, University of Bern 54179, Bern, Switzerland; 2 Institute of Geography, University of Bern 30322, Bern, Switzerland; 3 Oeschger Centre for Climate Change Research, University of Bern 27210, Bern, Switzerland; 4 Division of Molecular Bacterial Epidemiology and Infectious Diseases, Institute of Veterinary Bacteriology, Vetsuisse Faculty, University of Bern 30398, Bern, Switzerland; Medical College of Wisconsin, Milwaukee, Wisconsin, USA

**Keywords:** dairy calves, antimicrobial resistance, antimicrobial use, weather, calf pen, hutch

## Abstract

The objective of this study was to describe the non-linear relationships between antimicrobial resistance to at least one tested antimicrobial (AMR) or multidrug resistance (MDR; resistance to at least three antimicrobial drug classes) of *Escherichia coli* isolates from calf feces and from the calf environment on dairy farms, and farm-level atmospheric variables, as well as selected calf- and herd-level factors. Data sets of 601 fecal isolates and 777 environmental isolates, collected from 57 dairy farms on 204 and 212 sampling dates, respectively, were combined with atmospheric data closest to each farm (monthly average of values prior to each sampling date). By employing random forest models, we found that the maximum age of the sampled calves in the pooled fecal sample showed the highest importance for the outcome MDR in the calf fecal data set. For the outcome AMR in the calf data set and for both outcomes in the environment data set, all the atmospheric variables had the highest importance. In *E. coli* from the calf environment, AMR was negatively associated with the average, maximum, and minimum temperature in the month prior to sampling. In both the calf and environment data sets, both AMR and MDR were positively associated with increasing deviation from historical temperature. These findings warrant confirmation in larger data sets.

**IMPORTANCE:**

The dynamics of expansion and transmission of resistant bacterial populations and of antimicrobial resistance (AMR) genes within as well as between human, animal, and environmental reservoirs are complex. An association between average minimum ambient temperature and AMR rates in selected human pathogenic bacteria has recently been reported. The present study highlights the relative importance of atmospheric variables for the outcomes AMR and multidrug resistance (MDR), as well as associations between atmospheric variables and the AMR outcomes in two *Escherichia coli* data sets from calves’ fecal and environmental samples from Swiss dairy farms. In both the calf and environment data sets, both AMR and MDR were positively associated with increasing deviation from historical temperature. In the face of rapid anthropogenic global warming, these findings warrant further research on the association between AMR in livestock species and atmospheric variables.

## INTRODUCTION

The development and spread of antimicrobial resistance (AMR) represent a major threat to global health and food security (1). Antimicrobial use (AMU) is well recognized as a major contributor to AMR selection, both in humans and livestock populations. However, the dynamics of expansion and transmission of resistant bacterial populations and of AMR genes within as well as between the human, animal, and environmental reservoirs are complex and likely influenced by a variety of factors (2). Globally, such factors include hygiene and infection prevention and control, human and animal movements, human and animal waste management, and food hygiene (2–4). Often overlooked factors include atmospheric variables, such as temperature, which have been associated with AMR rates in human pathogenic isolates at national and international levels (3, 5–7). Additionally, there is evidence pointing toward such an association at the animal-herd level. On dairy farms, season and average monthly temperature have been associated with odds of AMR outcomes (e.g., AMR to specific antimicrobial drugs) in *Escherichia coli* from fecal samples (8, 9). Seasonal variations in disease incidence and AMU have also been reported and were hypothesized to be associated with a combination of changes in management practices, stocking density, and variations in atmospheric conditions such as ambient temperature and humidity (10–12). Atmospheric conditions may be associated with the survival or replication rate of bacteria in the cattle environment, and as such, with the risk of infection by pathogenic or opportunistic bacteria (11), as well as with the risk of inoculation and gut colonization of the animals with generic or specific bacterial strains (13). Therefore, when evaluating the association between AMR outcomes and factors such as herd-level AMU, atmospheric variables should be investigated as potentially confounding factors. This is especially important in a rapidly changing global climate, where AMR and anthropogenic global warming have the potential for non-linear and catastrophic interactions.

In a previous repeated cross-sectional study on 57 Swiss dairy farms, our group identified various sample- and herd-level factors (using mixed-effects logistic regression models) as potentially associated with AMR (resistance to at least one tested antimicrobial) and multidrug resistance (MDR; resistance to at least three antimicrobial drug classes) in *E. coli* isolates from pooled calf feces and from calf environmental swabs (V. Bernier Gosselin et al., submitted for publication; R. Zufferey et al., unpublished data). Moreover, we identified important variations over a one-year period in the number of antimicrobial classes to which the isolates showed resistance. We set out to investigate whether atmospheric variables may play a role in these variations. However, analytical challenges may arise when studying the association between AMR outcomes and potentially related factors in dairy herds. Outcomes and independent variables are often measured at different levels (i.e., bacterial isolate level vs animal and herd levels, e.g., calf age and herd-level AMU) and at different time and spatial resolutions (e.g., measured once vs monthly or yearly). Many investigated factors may be associated with one another, and plausible interactions in such data structures are difficult to identify. The important co-variability resulting from the inclusion of multiple atmospheric variables for investigation represents an additional analytical challenge. Nevertheless, the high spatial and temporal resolution of the available atmospheric data was well suited to this investigation.

The objective of the present study was to combine the existing cross-sectional study data with atmospheric data in order to gain a better understanding of the non-linear relationships between isolate-level AMR or MDR, farm-level atmospheric variables (monthly average of values prior to each sampling date), and selected calf- and herd-level factors, including AMU indices for the month prior to each sampling date.

## RESULTS

### Description of data sets

#### Fecal *E. coli* data set

The “fecal” data set was composed of 601 commensal *E. coli* isolated from pooled fecal samples collected from the calves’ rectum. The pools included up to three calves without a history of antimicrobial therapy aged between 27 and 67 days. One pooled sample per visit was collected upon up to four visits per farm, conducted at approximately 3-month intervals on 57 dairy farms, and three *E. coli* isolates per sample were characterized. Isolates were clustered at the sample and farm levels. After exclusion of visits without eligible calves and of missing *E. coli*, the 601 isolates of the final data set originated from a total of 204 farm visits. The proportions (95% CI) of isolates showing AMR (based on epidemiological cutoff [ECOFF] values) and MDR were 47.1% (43.0–51.2%) and 24.1% (20.8–27.8%), respectively. Independent variables that were retained in the data set based on an association (*P* value < 0.20, in mixed-effect logistic regression models) with either AMR or MDR are listed in Table 1.

**TABLE 1:** Calf- and farm-level independent variables included (indicated by an “x”) in the multivariable models for AMR (resistance to at least one tested antimicrobial, based on epidemiological cutoff values) and MDR (resistance to at least three antimicrobial classes), respectively, in the fecal and environmental data sets, with their respective distribution^*i*^

Item	Variable	*n* (%)	Reference category	Fecal	Environmental
1	Annual average milk production per cow ≥8,500 L	30/57 (52.6)	<8,500		x
2	Annual average milk somatic cell count >150,000 cells/mL	15/49 (30.6)	≤150,000		x
3	Housing of lactating cows in tie stalls	21/57 (36.8)	Free stall	x	x
4	Calving pen used for sick cows	30/57 (52.6)	No/no calving pen	x	
5	Calves moved out of the birth area >1 h after birth	37/57 (64.9)	≤1 h	x	x
6	Contact of calves 0–30 days with older cattle or their feces	32/57 (56.1)	No		x
7	Average number of calves within a pen	–^*a*^	–	x	x
8	Group composition dynamic	37/57 (64.9)	Constant	x	x
9	Main calf diet: milk replacer or milk replacer mixed with fresh milk	16/57 (28.1)	Fresh milk	x	x
10	Feeding waste milk with antimicrobial residues	30/57 (52.6)	No	x	x
11	Use of separate equipment for feed and manure	46/57 (80.7)	No		x
12	Use of group-specific equipment for pre-weaned calves	12/57 (21.1)	No	x	x
13	Veal calves fattened on the farm	8/56 (14.3)	No	x	
14	Number of introduced animals in the study year	–^*b*^	–	x	
15.1	Number of farms of origin of introduced animals: ≥2	38/53 (71.7)	0–1	x	
15.2	Number of farms of origin of introduced animals	–^*c*^	–		x
16	Source of introduced animals	–^*d*^	–	x	
17	Dog on the farm	30/53 (56.6)	No	x	x
18	Rodent control	21/53 (39.6)	No		x
19	Bird control	5/53 (9.4)	No		x
20	Incidence of pneumonia in calves 0–30 days old >10%	9/53 (17.0)	≤10%	x	
21	Incidence of pneumonia in calves 30 days—weaning >10%	9/52 (17.3)	≤10%	x	x
22	Incidence of diarrhea in calves 0–30 days old >15%	13/53 (24.5)	≤15%	x	
23	Incidence of diarrhea in calves 30 days–weaning >15%	5/52 (9.6)	≤15%	x	
24	Herd-level systemic antimicrobial use in the month prior to sampling	–^*e*^	No	x	x
25	Antimicrobial use in calves in the month prior to sampling	–^*f*^	No	x	x
26	Exposure of sampled calves to colostrum from cows treated with dry-off antimicrobials	83/204 (40.7)	No	x	
27	Maximum age of the calves in the pooled sample	–^*g*^	–	x	
28	Minimum age of the calves in the pooled sample	–^*h*^	–	x	

^*a*^ Average number of calves per group, 1–3: 22/57 (38.6%); 3.5–6: 26/57 (45.6%); and 6.5–10: 9/57 (15.8%).

^*b*^ Number of introduced animals, 0: 8/53 (15.1%); 1–4: 10/53 (18.9%); and >5: 35/53 (66.0%).

^*c*^ Number of farms of origin of introduced animals, 0: 8/53 (15.1%); 1: 7/53 (13.2%); 2–4: 14/53 (26.4%); and 5–40: 24/53 (45.3%).

^*d*^ Source of introduced animals, none: 8/53 (15.1%); directly from the farms: 33/53 (62.3%); and ≥1 animal over livestock market: 12/53 (22.6%).

^*e*^ Herd-level systemic antimicrobial use: fecal data set, yes: 141/204 visits (69.1%); environmental data set, yes: 148/212 visits (69.8%).

^*f*^ Antimicrobial use in calves: fecal data set, yes: 63/204 visits (30.9%); environmental data set, yes: 64/212 visits (30.2%).

^*g*^ Maximum age of the calves (continuous variable): median 43, interquartile range 36–53.5 days.

^*h*^ Minimum age of the calves (continuous variable): median 37, interquartile range 33–46 days.

^*i*^ Variables were selected for each data set based on an association with either AMR or MDR (*P* value < 0.20, in unconditional mixed-effect logistic regression models with sample or sampling date nested within herd as random effects). Denominators indicate the number of farms or visits (items 26–28) for which data for the variable were available.

#### Environmental *E. coli* data set

The “environmental” data set was composed of 777 *E. coli* isolated from swabs from the calf housing environment (walls or railings). Four swabs per visit were collected upon four visits per farm as described above, and one *E. coli* isolate per swab was characterized. Isolates were clustered at the visit and farm levels. The 777 isolates of the final data set originated from a total of 212 farm visits. The proportions (95% CI) of isolates showing AMR and MDR were 39.4% (35.9–42.9%) and 23.6% (20.6–26.7%), respectively. Independent variables that were retained in the data set based on an association (*P* value < 0.20, in mixed-effect logistic regression models) with either AMR or MDR are listed in Table 1.

#### Atmospheric variables

For the farm location and sampling timings presented here, the average temperature during the 30 days prior to the 212 sampling dates had a median of 9.0°C (interquartile range [IQR]: 2.9–15.5; range: −1.2 to 23.4). For the maximum temperature over the same period, the median was 21.5°C (IQR: 14.8–28.0; range: 7.8–36.8). The minimum recorded temperature had a median of −1.2°C (IQR: −6.2 to 4.9; range: −13.3 to 14.2). The 30-day average recorded precipitation had a median of 2.4 mm (IQR: 1.7–3.5; range: 0.4–7.7). The deviation from the historical (1981–2010) average temperature and precipitation for the same period had a mean (SD) of 2.0 (1.1)°C and −0.6 (1.2) mm, respectively.

### Ranking of variables by relative importance

#### Fecal *E. coli* data set

**Fig 1:**
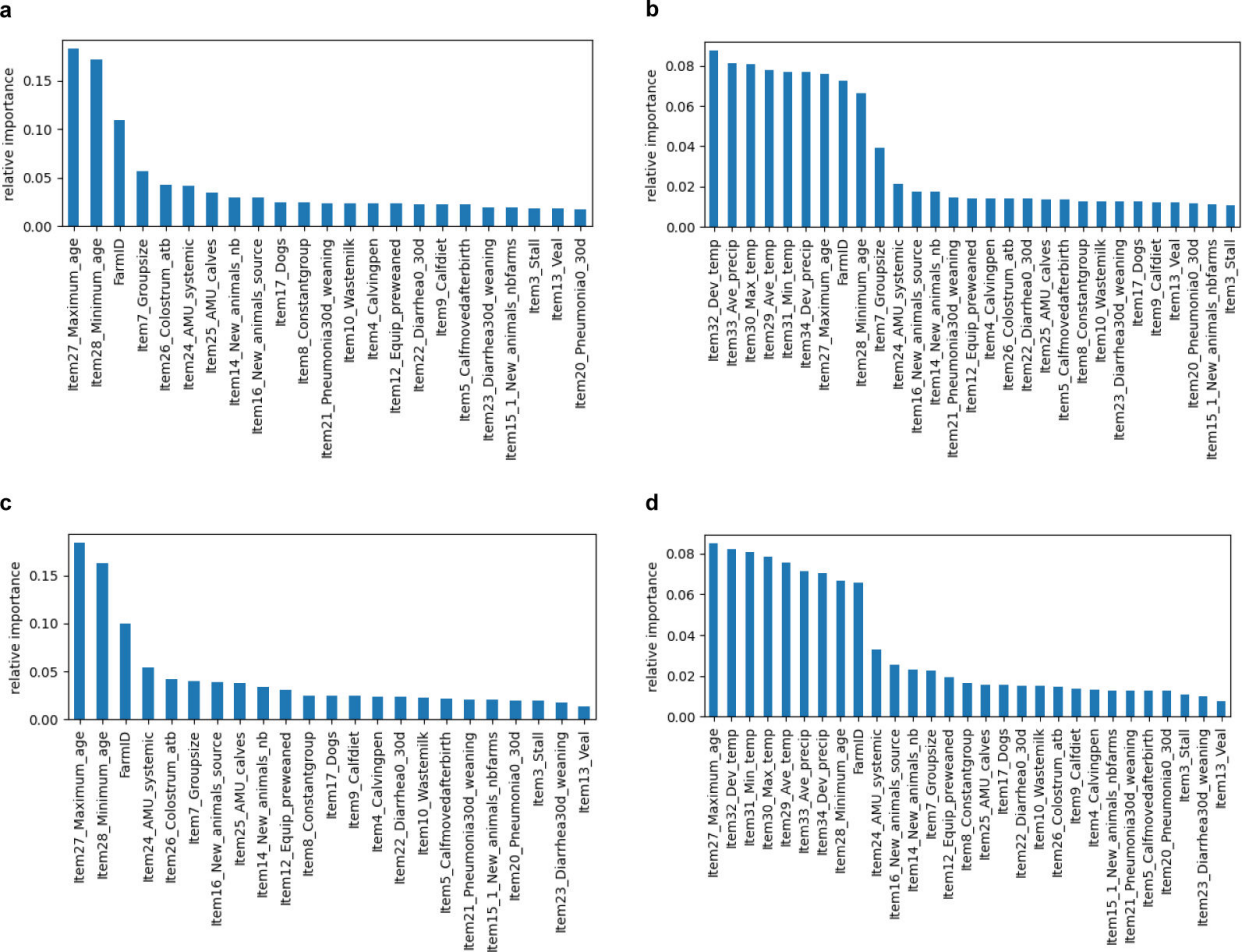
Ranking by importance of selected variables for the outcomes of antimicrobial resistance (AMR; resistance to at least one tested antimicrobial, based on epidemiological cutoff values) (**a and b**) and multidrug resistance (MDR; resistance to at least three antimicrobial drug classes) (**c and d**) in the data set of calf fecal *Escherichia coli* isolates, without atmospheric variables (**a and c**) and with atmospheric variables (**b and d**).

Figure 1 shows the results, for the outcomes AMR and MDR, of the ranking of variables listed in Table 1, with and without atmospheric variables. For both outcomes, in the models without atmospheric variables, the most important variables were those related to the sampled calves’ age and farm. In the MDR model, including atmospheric variables, maximum calf age in the pool was also the most important parameter, followed by all the atmospheric variables, whereas in the AMR model, the atmospheric variables had the highest importance, followed by calf age and farm variables.

#### Environmental *E. coli* data set

**Fig 2:**
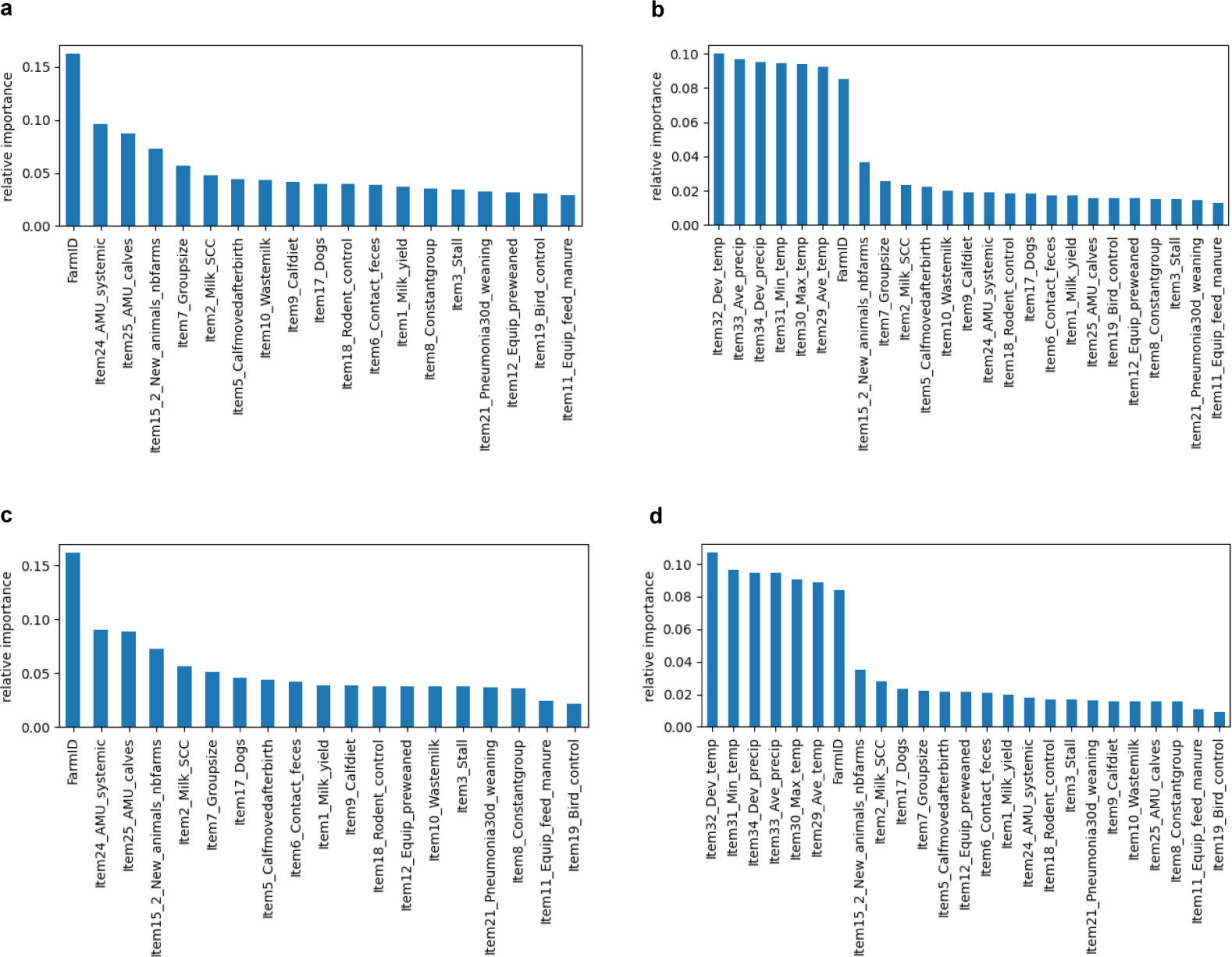
Ranking by importance of selected variables for the outcomes antimicrobial resistance (AMR; resistance to at least one tested antimicrobial, based on epidemiological cutoff values) (**a and b**) and multidrug resistance (MDR; resistance to at least three antimicrobial drug classes) (**c and d**) in the data set of environmental *Escherichia coli* isolates, without atmospheric variables (**a and c**) and with atmospheric variables (**b and d**).

Figure 2 shows the results, for the outcomes AMR and MDR, of the ranking of variables listed in Table 1, with and without atmospheric variables. In both models without atmospheric variables, farm is the most important variable. In the models including the atmospheric variables, all atmospheric variables rank above the variable farm, although the latter remains relatively important. For both the fecal and environmental data sets, the deviation from the historical temperature always ranks highest among the atmospheric variables, whereas the order of the remaining variables slightly differs between the graphs.

### Partial dependence plots

#### Fecal *E. coli* data set

**Fig 3:**
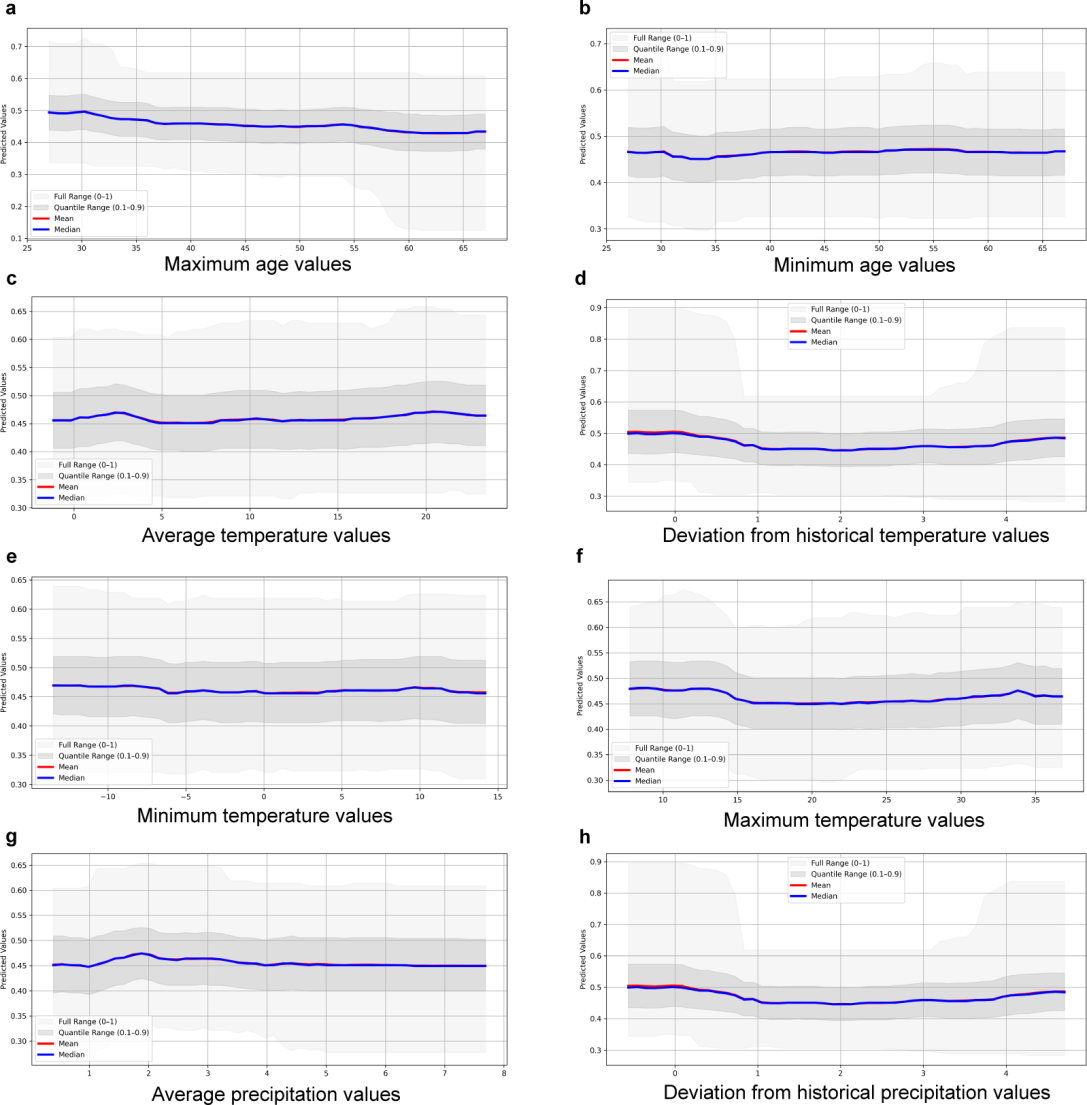
Graphs of the predicted change (mean and median) in the probability of antimicrobial resistance (AMR; resistance to at least one tested antimicrobial, based on epidemiological cutoff values) in an *Escherichia coli* isolate from a calf fecal sample, in relation to changes in the predictors (**a**) maximum calf age, (**b**) minimum calf age, (**c**) average temperature, (**d**) deviation from historical temperature, (**e**) minimum temperature, (**f**) maximum temperature, (**g**) average precipitation, and (**h**) deviation from historical precipitation.

**Fig 4:**
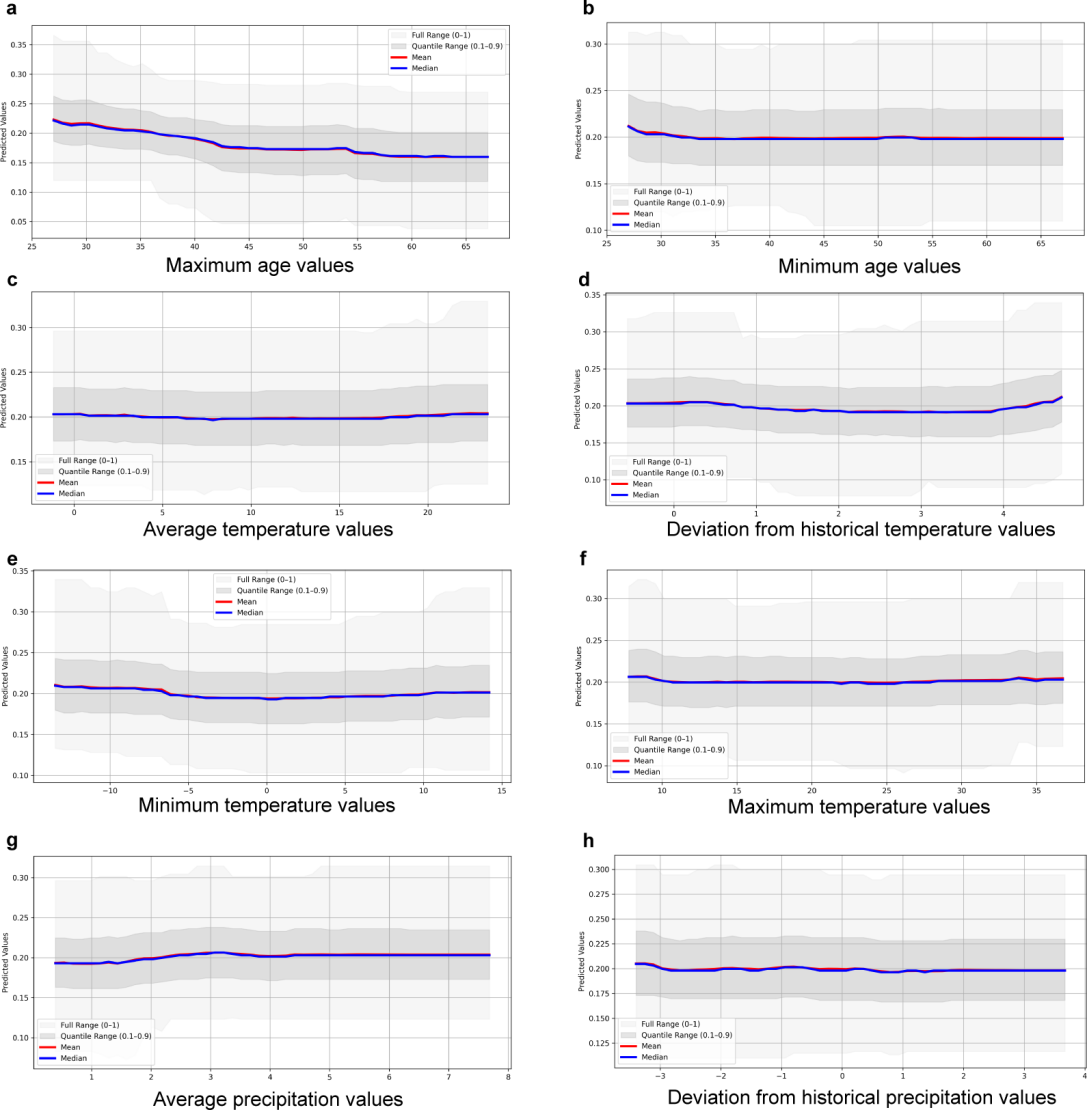
Graphs of the predicted change (mean and median) in the probability of multidrug resistance (MDR; resistance to at least three antimicrobial drug classes, based on epidemiological cutoff values) in an *Escherichia coli* isolate from a calf fecal sample, in relation to changes in the predictors** (a**) maximum calf age, (**b**) minimum calf age, (**c**) average temperature, (**d**) deviation from historical temperature, (**e**) minimum temperature, (**f**) maximum temperature, (**g**) average precipitation, and (**h**) deviation from historical precipitation.

Based on their relative importance, the atmospheric variables as well as maximum and minimum calf age were selected for the investigation of the direction of influence on the outcome variables. The graphs showing the mean and median of the models for AMR and MDR are presented in Fig. 3 and 4, respectively. The predicted probability of AMR and MDR decreased with increasing maximum age of the sampled calves in the pool, whereas the direction of effect of minimum age did not follow a clear trend and differed between AMR and MDR. The variables average, maximum, and minimum temperature, as well as deviation from historical temperature, followed a similar pattern with increased probability of AMR and MDR at low and high values. The highest magnitude of change in predicted probability was observed for AMR with the variables maximum temperature and deviation from historical temperature. To investigate whether these findings were driven by resistance to a specific antimicrobial drug, partial dependence plots for the most common resistances in the data sets (i.e., ampicillin, sulfamethoxazole, and tetracycline) were also generated. A pattern similar to that observed for total AMR, with increased probability of resistance at low and high values, was only observed with the variable deviation from historical temperature (see Fig. S1 at https://doi.org/10.48620/96179 [14]).

#### Environmental *E. coli* data set

**Fig 5:**
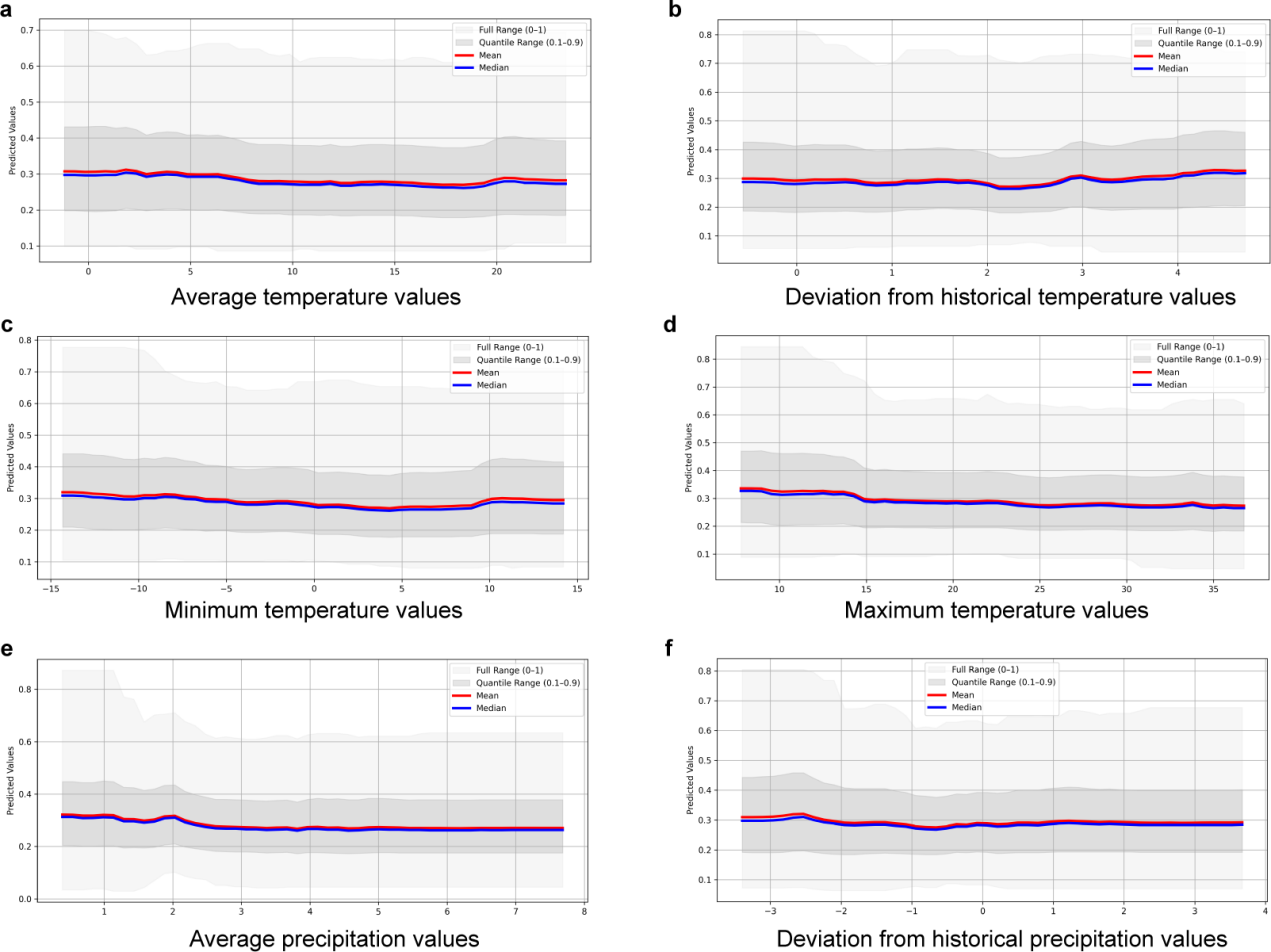
Graphs of the predicted change (mean and median) in the probability of antimicrobial resistance (AMR; resistance to at least one tested antimicrobial, based on epidemiological cutoff values) in an *Escherichia coli* isolate from a calf environmental swab sample, in relation to changes in the predictors (**a**) average temperature, (**b**) deviation from historical temperature, (**c**) minimum temperature, (**d**) maximum temperature, (**e**) average precipitation, and (**f**) deviation from historical precipitation.

**Fig 6:**
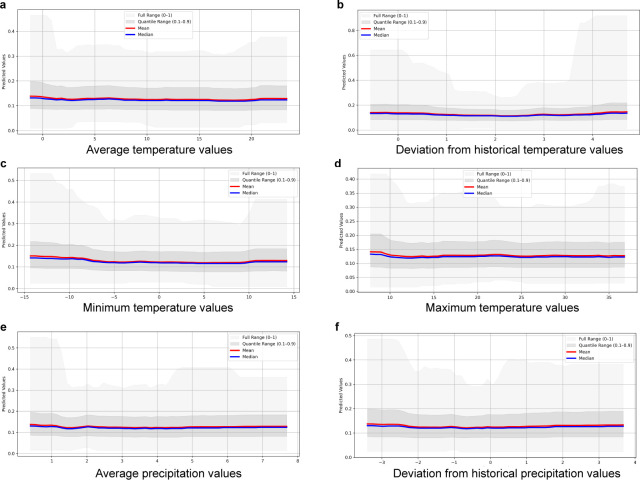
Graphs of the predicted change (mean and median) in the probability of multidrug resistance (MDR; resistance to at least three antimicrobial drug classes, based on epidemiological cutoff values) in an *Escherichia coli* isolate from a calf environmental swab sample, in relation to changes in the predictors (**a**) average temperature, (**b**) deviation from historical temperature, (**c**) minimum temperature, (**d**) maximum temperature, (**e**) average precipitation, and (**f**) deviation from historical precipitation.

The graphs showing the mean and median of the models of the direction of influence of atmospheric variables for AMR and MDR are presented in Fig. 5 and 6, respectively. The variables average, maximum, and minimum temperature, as well as average precipitation, followed a similar trend of decreasing probability of AMR with increasing values. The graph for deviation from historical temperature showed an increasing probability of AMR starting at values of >2°C above normal. For MDR, only the deviation from historical temperature showed a pattern of increased probability at low and high values (similar to that observed for fecal *E. coli*). For resistance to ampicillin, sulfamethoxazole, and tetracycline, a pattern similar to that observed for total AMR was observed with the variables minimum temperature and deviation from historical temperature (see Fig. S2 at https://doi.org/10.48620/96179 [14]).

## DISCUSSION

In the present study, we evaluated the relationships between AMR and MDR of generic *E. coli* isolates from calf feces and calf environment and atmospheric variables, sample-level factors (fecal data set only), and farm-level factors. In the models excluding atmospheric variables, the most important variables associated with the outcomes AMR and MDR were consistent with previous reports, including sampled calf age (fecal data set) (15–17), herd-level AMU indices (environmental data set) (18), and farm-specific effect (both data sets). The latter effect could represent a combination of multiple management (measured and unmeasured) factors and indicates that it might be difficult to predict AMR outcomes based on measured individual factors. Interestingly, when including atmospheric variables, these ranked higher in importance than all other variables, except for maximum calf age in the MDR model. The comparison of the models with and without atmospheric variables revealed that while associations between herd variables and AMR outcomes may be found in models omitting atmospheric variables, the most important variables might actually be missing in the reduced models. When AMR outcomes vary at the farm level over time, the strength of association with the outcome might be weaker for a herd management variable measured once for the entire study duration, for instance, the introduction of animals from multiple farms, than for a variable measured contemporarily to the outcome, such as sampled calf age. Although the lower importance of most herd-level variables could be attributed to the fact that they were only measured once, AMU indices were estimated for the month prior to each sampling date and were nevertheless less important than atmospheric variables. These results are in line with those of a previous study on fecal *E. coli* from slaughtered beef cattle, in which the reported effect of season on the prevalence of AMR outcomes was greater than the production system (cattle raised with or without antibiotics, although specific treatment history was unknown) (19). It should be emphasized that the calves sampled for the present study had not been treated with antimicrobials. Of note, due to the AMU data distributions in the present study, the two AMU indices retained for the models were dichotomized, with approximately 30% and 70% of observations having no herd-level systemic AMU and no AMU in calves, respectively, in the month prior to sampling. While this is a plausible occurrence in small dairy farms such as the farms enrolled in the present study, different results could have been obtained on larger farms and with AMU as a continuous variable. Another observation from the comparison of models with and without atmospheric variables using the environmental data set was that systemic AMU ranked lower (after multiple herd-level variables) in the models including atmospheric variables compared to the models without. This might indicate a confounding effect of atmospheric variables, where a portion of the association between AMU indices and AMR outcomes is attributable to an association of AMU indices and AMR outcomes with atmospheric variables and is therefore reduced when the latter are accounted for in the models. Indeed, associations between herd-level systemic AMU in the month prior to sampling and average temperature (*P* value = 0.07), minimum temperature (*P* value = 0.001), and deviation from historical temperature (*P* value = 0.04) had also been identified in unconditional mixed-effects logistic regression models (data not shown). The relative importance and the possibility of confounding support the need to include atmospheric variables in future epidemiological investigations of AMR outcomes in farm environments and in the fecal flora of animals without prior or recent antimicrobial treatment. Failure to account for potential confounding factors could result in biased measures of association between investigated factors and AMR outcomes.

Among the evaluated atmospheric variables, deviation from historical temperature ranked highest in importance. The magnitude of change in AMR outcome probability varied between outcomes and data sets, so that none of the temperature variables (average, minimum, or maximum) consistently appeared to be a better predictor than the others. In previous studies on the association between climate and AMR in human pathogens, minimum temperature was selected based on its potential role in environmental persistence and transmission (5, 6). In another study of *E. coli* from dairy farm environments, average temperature was selected to be evaluated in association with AMR outcomes (8). In the latter study, an association between tetracycline resistance and average monthly rainfall was additionally reported (in a model also including farm-level AMU). In the present study, for most data set and AMR outcome combinations, average precipitation and deviation from historical precipitation did not show a clear trend. Together, the results of the present and previous studies suggest that until a single meteorological variable can be consistently considered as the most relevant, future investigations of associations with AMR outcomes at the farm level should include all six evaluated meteorological variables, especially deviation from historical temperature in the context of global warming.

In previous studies, factors hypothesized to contribute to an association of AMR outcomes with temperature on a global scale included increasing temperature favoring bacterial growth, horizontal gene transfer, and transmission between hosts, as well as from the environment to hosts (5, 6, 20, 21). On the other hand, temperature increases might represent a stress for *E. coli*, and survival under different ambient conditions may differ between strains (22). In the present study, *E. coli* isolates from calf fecal commensal flora and from calf environment were collected on the same day. In both data sets, the probability of both AMR outcomes increased with increasing deviation from historical temperature. Yet, the direction of associations between AMR outcomes and the remaining temperature variables diverged between both data sets. In the fecal data set, a higher probability of AMR was observed at both low and high values of maximum temperature. This could be explained by changes in the calf gut homeostasis at temperatures outside the calf thermoneutral zone, such as local immunity and microbiota (23), potentially associated with calf fecal *E. coli* populations and resistant subpopulations (20, 24). Further research on the processes involved and the potential role of their mitigation (i.e., thermal stress prevention) is warranted. Conversely, in the environmental data set, the probability of AMR tended to decrease with increasing values of the temperature variables (minimum, maximum, and average). This could reflect that the environmental *E. coli* are more directly exposed to ambient conditions (as opposed to fecal, i.e., intestinal *E. coli*), favoring or not their survival, fitness, and growth on calf housing environmental surfaces (13, 22). In this regard, an important limitation of the present study is that the atmospheric data obtained from the nearest meteorological information may not always accurately reflect the conditions in the farm environment (25). Whether the sampled environmental surfaces were located inside the main barn or outdoors was not recorded, which might have affected the observed association differentially depending on the calf housing type in use between farms and between visits on the same farm. Similarly, other factors potentially affecting the survival or fitness of bacterial strains on environmental surfaces, such as surface material and cleaning and disinfection frequency (26), were not taken into account.

Recent study reports have highlighted an association between atmospheric variables such as average minimum ambient temperature and AMR rates in selected human pathogenic bacteria (5–7). In contrast to the latter studies using national AMR surveillance and weather data, AMR data in our data set were from commensal *E. coli* in calves and in their housing environment, obtained from a limited number of farms distributed over a relatively small geographical area. The atmospheric information was extracted from the grid box in which each farm was located. We acknowledge that this entails uncertainty regarding catching microclimates at the farm location (such as hills, pits, or local vegetation impacts) as well as uncertainties regarding possible indoor conditions at farms and the free-air conditions represented in the atmospheric data sets. Nevertheless, these data represent an unprecedented level of spatial resolution and contextual relevance for farm-level atmospheric assignment in studies of this kind, offering a substantial improvement over previous approaches that relied on coarser grids or distant weather stations. Hence, we hypothesized that the high spatial and temporal resolution of the atmospheric data in our data set might allow for the detection of an association between atmospheric variables and AMR outcomes. Atmospheric variables could be associated with geographical location and agricultural zone (e.g., mountain), which could, in turn, be associated with other herd-level variables such as herd size and management practices. Monthly atmospheric variables could also be associated with variations in disease incidence, and consequently with monthly AMU (10–12). The atmospheric variables average, minimum, and maximum temperature, as well as deviation from historical temperature, are also highly correlated with one another. The selected modeling approach made it possible to take such associations between multiple variables into account. Deviation from historical temperature, however, is not associated with seasonal variations in the temperature variables, thus reducing the co-variability with seasonally changing factors such as stocking density and other farm management practices. In two previous studies in fattening beef cattle, seasonal variations were reported to be associated with the total counts of generic *E. coli* in fecal samples, as well as counts and prevalence of *E. coli* showing resistance to tetracycline, trimethoprim-sulfamethoxazole, and third-generation cephalosporin, with increases observed in spring-summer and summer-fall, respectively (19, 27). In another study investigating extended-spectrum β-lactamase (ESBL)-producing *E. coli* in samples from fecally contaminated calf environments on 53 dairy farms, the odds of farm-level ESBL-producing *E. coli* positivity were associated with monthly average temperature, which was hypothesized to be attributable to a lower fitness of resistant strains at lower temperatures (8). This is opposite to the observed association with AMR outcomes in generic *E. coli* from the calf environment in the present study. Part of this difference might be explained by the different isolate selection protocol and the type of samples, where in our study, the sampling of fresh or dry feces from the calf pen walls was avoided. Additionally, most farms in the former study were located within a 50 × 50 km area (8). Although each farm of the present study was sampled at 3-month intervals (with visits on all farms distributed over time) instead of monthly, their distribution in various Swiss cantons and at diverse altitudes resulted in a larger number of observation points and broader variation in atmospheric variables. The implications of these findings are twofold. On the one hand, future observational studies on AMR outcomes on dairy farms should consider, at the design and analysis stages, geographical and seasonal variations (between and within farms, respectively) as potentially affecting the outcomes and observed associations (e.g., between AMU and AMR). On the other hand, on a larger scale, further research on the association between AMR in animal species from national surveillance data and atmospheric variables, including long-term temporal trends, is warranted, especially in the face of rapid anthropogenic global warming, with Switzerland warming considerably faster than the global average (28). Though disease prevention measures and prudent AMU should remain a cornerstone of the global fight against AMR (29), a better understanding of the role of atmospheric variables in the development and spread of AMR is needed.

### Limitations

The analyses of the present study were based on data sets of generic *E. coli* from calf feces and calf environment collected over the course of a repeated cross-sectional study on a convenience sample of 57 Swiss dairy farms. Although the respective data sets comprised 601 and 777 isolates, the fecal and environmental isolates were clustered within sample and within sampling date, respectively, in addition to within farm. However, the selected modeling approach did not allow for a three-level data structure. As a result, the analyses were conducted on a random selection of one isolate per sample or sampling date, on sets of 204 or 212 isolates, respectively, for a total of 10,000 repetitions for increased robustness. Nevertheless, the small sample size of the data set in general and class imbalances for MDR specifically resulted in a low level of mean accuracy across the 10,000 random runs of around 0.55/0.56 for predicting AMR and 0.75/0.73 for MDR in the calf feces and calf environment data sets, where the precision for the latter is only 0.11/0.05, respectively. These results suggest a large amount of false positive predictions for MDR. That being said, the focus of this study was to explore the non-linear importance of predictors rather than to create the most performant prediction model. Nevertheless, the results are suggestive of an association, which should be confirmed on larger data sets of generic *E. coli* from calf feces and farm environments. The trends in probability of resistance to ampicillin, sulfamethoxazole, or tetracycline in relation to changes in temperature variables did not fully mirror those of total AMR. The small sample size combined with a low prevalence of resistance to specific antimicrobial drugs (e.g., quinolones) precluded a more detailed analysis for each tested drug resistance. Associations of atmospheric variables with specific resistances could potentially give a hint to the involved mechanisms, for example, increased mutagenesis or increased transfer of mobile genetic elements. A broader selection of resistance outcomes, such as resistance to individual or groups of antimicrobial drugs, should therefore be included in future, larger studies. Finally, the inclusion in future studies of samples from regions experiencing smaller temperature variations (e.g., tropics) might be considered, though other factors such as differences in farm management and national AMU policies should additionally be taken into account.

### Conclusion

In *E. coli* from pooled calf feces, MDR was negatively associated with the maximum age of sampled calves in the pool. In *E. coli* from the calf environment, AMR was negatively associated with the average, maximum, and minimum temperature in the month prior to sampling. In both data sets, both AMR outcomes were positively associated with increasing deviation from historical temperature. With the exception of maximum age in the calf fecal data set, atmospheric variables showed the most relative importance for AMR outcomes in the chosen models. These initial, but consequential findings warrant confirmation in larger data sets.

## MATERIALS AND METHODS

### Study population and variable selection

The two *E. coli* data sets (calves’ fecal and environmental samples) originated from a repeated cross-sectional study. A convenience sample of 57 dairy farms located in the Swiss cantons of Bern, Fribourg, Neuchâtel, Solothurn, Jura, Lucerne, Aargau, Vaud, Basel, and Zurich was enrolled. The farms were visited at approximately 3-month intervals for up to four visits between January 2022 and February 2023. The median number of lactating cows per herd, averaged over all visits, was 28 (interquartile range: 22.3–37; range: 11.3–61.3). Upon each visit, one pooled fecal sample from the rectum of up to three calves (27–67 days old) without antimicrobial treatment since birth, and four gauze swabs from the calf housing environment were collected and placed in labeled polypropylene tubes. Environmental swabs were collected over the surface of pen walls (30 cm height by 150 cm length, when available) or railings, while avoiding smears of fecal material (18). In the laboratory, Müller-Hinton broth (30 mL) was added to each tube. The samples were then spread onto Enterobacterales-selective agar (BROLAC [Thermo Fisher Scientific, Waltham, USA] or MacConkey [bioMérieux, Marcy-l’Étoile, France]) using either a cotton swab (fecal samples) or a 100 μL inoculation loop following an incubation period of 18–24 h at 37°C (environment samples), and incubated at 37°C for 24 h. Three *E. coli* colonies per fecal sample and one per environmental swab sample were randomly selected. Species identification was confirmed as *E. coli* by MALDI-TOF mass spectrometry (VITEK MS bioMérieux). Susceptibility to 15 antimicrobial drugs was determined by broth microdilution following EUCAST recommendations, using Sensititre EUVSEC3 test plates (Thermo Fisher Scientific). Resistance was defined based on ECOFF values (30), and MDR was defined as resistance to at least three antimicrobial drug classes. Herd characteristics and management practices were recorded by questionnaire-based interviews. Antimicrobial prescription data were obtained, with farmers’ and veterinarians’ written consent, from the Swiss Information System on Antibiotics in Veterinary Medicine (IS-ABV) and were summarized at the herd level for the month prior to each sampling. The sample- and herd-level variables to be included in each data set, listed in Table 1, were selected based on an association (*P* value < 0.20) with one of the outcomes (AMR, MDR, or both) by means of mixed-effect logistic regression models, with sample or sampling date nested within herd as random effects. Details of herd-level data cleaning and analysis are described elsewhere (Bernier Gosselin et al., submitted for publication).

### Atmospheric data

To extract the geographically closest atmospheric conditions for each farm and time stamp, we used two daily spatial data sets from MeteoSwiss for mean temperature (TabsD) and precipitation totals (RhiresD) (31, 32). These data sets are available for the period from 1 January 1961 to near real-time at a resolution of 1 × 1 km. The precipitation fields are generated using around 650 rain-gauge measurements in millimeters within Switzerland and from neighboring countries. They are based on the spatially interpolated monthly mean precipitation of a given day and spatial interpolations of relative anomalies (31). This data set does not differentiate between fluid and solid precipitation. The temperature fields represent the free-air temperature (°C) at 2 m above ground and are interpolated from approximately 90 homogeneous long-term station series using a deterministic analysis method with nonlinear vertical temperature profiles and non-Euclidean distance (33). These data sets are published free of charge by MeteoSwiss as “Open Data” with a CC-BY license (34). Both data sets are well-tested, trustworthy, maintained, and used in many scientific publications.

To assign weather information to a given farm, we extracted the atmospheric information from the grid box in which each farm was located. Besides using absolute daily mean values, we calculated daily mean anomalies (deviation) for each location with respect to the daily climatology (average) for the time period 1981–2010. Data were extracted for a 30-day period prior to each visit on the respective farms, and included minimum, maximum, and mean temperature during that period, mean deviation from the daily temperature climatology, 30-day mean precipitation, and mean deviation from the daily precipitation climatology. To simplify the text, 30-day mean deviation from the daily temperature climatology and 30-day mean deviation from the daily precipitation climatology are referred to as deviation from historical temperature and deviation from historical precipitation, respectively.

### Statistical analyses

For some herd-level categorical variables, values were missing for one farm (Table 1; Item 13), four farms (Items 15–20 and 22), five farms (Items 21 and 23), or eight farms (Item 2). These missing observations were assigned to a third (median) category. For the environmental data set including the variable with missing data for eight farms (average milk somatic cell count), the analyses were conducted before and after excluding the variable. There was no evidence for a bias resulting from the categorization of missing values (unchanged ranking of the remaining variables); therefore, the variable was kept in the models. In the calf fecal data set, the proportions of isolates showing AMR and MDR were 47.1% (283/601) and 24.1% (145/601), respectively. In the environmental data set, the proportions of isolates showing AMR and MDR were 39.4% (306/777) and 23.6% (183/777), respectively.

To perform the non-linear binary classification task, we used a well-established machine learning random forest (RF) model (35), where the label data or predictand (*y*) is the binary information of AMR or MDR, and the feature or predictor data (*X*) contains the farm-level management data and atmospheric variables. The principle of a RF model is to compute a large number of decision trees, each fitted with a random sub-selection of the initial data, to assess the average link of each predictor with the predictand and its importance compared to the others. This approach allows investigating the relative importance of each feature as well as the partial dependence of the label outcome on each individual feature. Relative feature importance quantifies the loss in predictive performance when this predictor is not included in the decision tree. A partial dependence plot shows how the average probability of observing a specific outcome changes with changes in a single predictor variable.

The fecal and environmental isolates were clustered within sample and within sampling date, respectively, in addition to within farm. However, the selected modeling approach did not allow for a three-level data structure. Consequently, the analyses were conducted on a random selection of one isolate per sample or sampling date, on sets of 204 or 212 isolates, respectively, for a total of 10,000 random repetitions for increased robustness. Each of the independent samples of *n* = 204 or *n* = 212 was then split into 80% for training and 20% for evaluation during model training. Additionally, to account for the clustering of isolates within farm, farm was included as a predictor variable. Note that the random selection did not control for equal amount of binary values in the label data, and as such, each random selection includes a different amount of 0 and 1 labels for the predictand. Although the focus of this study was to explore the non-linear importance of predictors rather than to create the most performant prediction model, we measured the skill of the RF prediction model with the commonly used metrics of recall, precision, and accuracy.

Following the initial analyses, partial dependence plots were additionally generated for outcomes of resistance to specific antimicrobial drugs in relation to changes in temperature variables. Based on a minimum prevalence of 20% in each data set, outcomes of resistance to ampicillin, sulfamethoxazole, and tetracycline were selected. In the calf fecal data set, the proportions of isolates showing resistance to ampicillin, sulfamethoxazole, and tetracycline were 24.1%, 35.9%, and 37.6%, respectively. In the environmental data set, the proportions of isolates showing resistance to ampicillin, sulfamethoxazole, and tetracycline were 23.3%, 32.2%, and 31.5%, respectively.

Data cleaning was performed using STATA 18.0 (StataCorp, College Station, TX, USA). Multivariable analyses were conducted using Python 3.11 with the scikit-learn library.

## Data Availability

Pseudonymized data are openly available on the University of Bern repository (https://doi.org/10.48620/96179). The code behind the random forest analysis can be found at https://github.com/martin-wegmann/AMRclimate.

## References

[1] World Health Organization (WHO). 2020. Antibiotic resistance. Available from: www.who.int/news-room/fact-sheets/detail/antibiotic-resistance

[2] Parker EM, Ballash GA, Mollenkopf DF, Wittum TE. 2024. A complex cyclical One Health pathway drives the emergence and dissemination of antimicrobial resistance. Am J Vet Res 85:ajvr.24.01.0014. doi:10.2460/ajvr.24.01.0014 38467112

[3] Allel K, Day L, Hamilton A, Lin L, Furuya-Kanamori L, Moore CE, Van Boeckel T, Laxminarayan R, Yakob L. 2023. Global antimicrobial-resistance drivers: an ecological country-level study at the human-animal interface. Lancet Planet Health 7:e291–e303. doi:10.1016/S2542-5196(23)00026-8 37019570

[4] Federal Office of Public Health and Federal Food Safety and Veterinary Office (FOPH and FSVO). 2022. Swiss antibiotic resistance report 2022. Usage of antibiotics and occurrence of antibiotic resistance in Switzerland. Available from: https://www.anresis.ch/wp-content/uploads/2022/11/BAG_Antibiotikaresistenz_INH_2022_RZ_Web.pdf

[5] MacFadden DR, McGough SF, Fisman D, Santillana M, Brownstein JS. 2018. Antibiotic resistance increases with local temperature. Nat Clim Chang 8:510–514. doi:10.1038/s41558-018-0161-6 30369964 PMC6201249

[6] McGough SF, MacFadden DR, Hattab MW, Mølbak K, Santillana M. 2020. Rates of increase of antibiotic resistance and ambient temperature in Europe: a cross-national analysis of 28 countries between 2000 and 2016. Euro Surveill 25:1900414. doi:10.2807/1560-7917.ES.2020.25.45.1900414 33183408 PMC7667635

[7] Kaba HEJ, Kuhlmann E, Scheithauer S. 2020. Thinking outside the box: association of antimicrobial resistance with climate warming in Europe - a 30 country observational study. Int J Hyg Environ Health 223:151–158. doi:10.1016/j.ijheh.2019.09.008 31648934

[8] Schubert H, Morley K, Puddy EF, Arbon R, Findlay J, Mounsey O, Gould VC, Vass L, Evans M, Rees GM, Barrett DC, Turner KM, Cogan TA, Avison MB, Reyher KK. 2021. Reduced antibacterial drug resistance and bla_CTX-M_ β-lactamase gene carriage in cattle-associated Escherichia coli at low temperatures, at sites dominated by older animals, and on pastureland: Implications for surveillance. Appl Environ Microbiol 87:e01468-20. doi:10.1128/AEM.01468-20 PMC8105006 33397699

[9] Duse A, Waller KP, Emanuelson U, Unnerstad HE, Persson Y, Bengtsson B. 2015. Risk factors for antimicrobial resistance in fecal Escherichia coli from preweaned dairy calves. J Dairy Sci 98:500–516. doi:10.3168/jds.2014-8432 25465547

[10] Federal Food Safety and Veterinary Office (FSVO). 2023. IS ABV Verschreibungen von Antibiotika für Tiere in der Schweiz 2022. Available from: https://www.blv.admin.ch/dam/blv/de/dokumente/tiere/tierkrankheiten-und-arzneimittel/tierarzneimittel/is-abv/jahresberichts-isabv-2022.pdf.download.pdf/PL_IS-ABV_Bericht_2022_DE.pdf. Retrieved 29 Oct 2025.

[11] Dachrodt L, Bartel A, Arndt H, Kellermann LM, Stock A, Volkmann M, Boeker AR, Birnstiel K, Do Duc P, Klawitter M, Paul P, Stoll A, Woudstra S, Knubben-Schweizer G, Müller KE, Hoedemaker M. 2022. Benchmarking calf health: assessment tools for dairy herd health consultancy based on reference values from 730 German dairies with respect to seasonal, farm type, and herd size effects. Front Vet Sci 9:990798. doi:10.3389/fvets.2022.990798 36213417 PMC9539667

[12] Martínez EP, van Rosmalen J, Jacobs J, Sanders P, van Geijlswijk IM, Heederik DJJ, Verbon A. 2023. Seasonality of antimicrobial use in Dutch food-producing animals. Prev Vet Med 219:106006. doi:10.1016/j.prevetmed.2023.106006 37647721

[13] Gautam R, Bani-Yaghoub M, Neill WH, Döpfer D, Kaspar C, Ivanek R. 2011. Modeling the effect of seasonal variation in ambient temperature on the transmission dynamics of a pathogen with a free-living stage: example of Escherichia coli O157:H7 in a dairy herd. Prev Vet Med 102:10–21. doi:10.1016/j.prevetmed.2011.06.008 21764472

[14] Bernier Gosselin V, Wegmann M, Zufferey R. 2026. Supporting data to associations of antimicrobial and multidrug resistance in Escherichia coli from the fecal flora and housing environment of calves on dairy farms with atmospheric variables. Available from: 10.48620/96179 PMC13203957 42059604

[15] Berge ACB, Atwill ER, Sischo WM. 2005. Animal and farm influences on the dynamics of antibiotic resistance in faecal Escherichia coli in young dairy calves. Prev Vet Med 69:25–38. doi:10.1016/j.prevetmed.2005.01.013 15899294

[16] Edrington TS, Farrow RL, Carter BH, Islas A, Hagevoort GR, Callaway TR, Anderson RC, Nisbet DJ. 2012. Age and diet effects on fecal populations and antibiotic resistance of a multi-drug resistant Escherichia coli in dairy calves. Agric Food Anal Bacteriol 2:162–174.

[17] Khachatryan AR, Hancock DD, Besser TE, Call DR. 2004. Role of calf-adapted Escherichia coli in maintenance of antimicrobial drug resistance in dairy calves. Appl Environ Microbiol 70:752–757. doi:10.1128/AEM.70.2.752-757.2004 14766551 PMC348837

[18] Bernier Gosselin V, Perreten V, Collaud A, Schüpbach-Regula G, Meylan M. 2025. Antimicrobial resistance in Escherichia coli isolates from the calf environment on Swiss dairy farms. Res Vet Sci 196:105893. doi:10.1016/j.rvsc.2025.105893 40957355

[19] Vikram A, Rovira P, Agga GE, Arthur TM, Bosilevac JM, Wheeler TL, Morley PS, Belk KE, Schmidt JW. 2017. Impact of "raised without antibiotics" beef cattle production practices on occurrences of antimicrobial resistances. Appl Environ Microbiol 83:e01682-17. doi:10.1128/AEM.01682-17 28887421 PMC5666148

[20] Mir RA, Weppelmann TA, Teng L, Kirpich A, Elzo MA, Driver JD, Jeong KC. 2018. Colonization dynamics of cefotaxime resistant bacteria in beef cattle raised without cephalosporin antibiotics. Front Microbiol 9:500. doi:10.3389/fmicb.2018.00500 29619015 PMC5871660

[21] Walsh TR, Weeks J, Livermore DM, Toleman MA. 2011. Dissemination of NDM-1 positive bacteria in the New Delhi environment and its implications for human health: an environmental point prevalence study. Lancet Infect Dis 11:355–362. doi:10.1016/S1473-3099(11)70059-7 21478057

[22] van Elsas JD, Semenov AV, Costa R, Trevors JT. 2011. Survival of Escherichia coli in the environment: fundamental and public health aspects. ISME J 5:173–183. doi:10.1038/ismej.2010.80 20574458 PMC3105702

[23] Yu Z, Cantet JM, Paz HA, Kaufman JD, Orellano MS, Ipharraguerre IR, Ríus AG. 2024. Heat stress-associated changes in the intestinal barrier, inflammatory signals, and microbiome communities in dairy calves. J Dairy Sci 107:1175–1196. doi:10.3168/jds.2023-23873 37730180

[24] Hinton M. 1985. The sub-specific differentiation of Escherichia coli with particular reference to ecological studies in young animals including man. J Hyg (London) 95:595–609. doi:10.1017/s0022172400060691 2419402 PMC2129571

[25] Schüller LK, Burfeind O, Heuwieser W. 2013. Short communication: Comparison of ambient temperature, relative humidity, and temperature-humidity index between on-farm measurements and official meteorological data. J Dairy Sci 96:7731–7738. doi:10.3168/jds.2013-6736 24140331

[26] Wang X, Gautam R, Pinedo PJ, Allen LJS, Ivanek R. 2014. A stochastic model for transmission, extinction and outbreak of Escherichia coli O157:H7 in cattle as affected by ambient temperature and cleaning practices. J Math Biol 69:501–532. doi:10.1007/s00285-013-0707-1 23864122

[27] Long NS, Wells JE, Berry ED, Legako JF, Woerner DR, Loneragan GH, Broadway PR, Carroll JA, Sanchez NCB, Fernando SC, Bacon CM, Helmuth CL, Smock TM, Manahan JL, Hoffman AA, Hales KE. 2022. Metaphylactic antimicrobial effects on occurrences of antimicrobial resistance in Salmonella enterica, Escherichia coli and Enterococcus spp. measured longitudinally from feedlot arrival to harvest in high-risk beef cattle. J Appl Microbiol 133:1940–1955. doi:10.1111/jam.15691 35766106 PMC9546201

[28] Federal Office of Meteorology and Climatology MeteoSwiss. Climate change. Available from: https://www.meteoswiss.admin.ch/climate/climate-change.html. Retrieved 2121 AprApril 2026. Accessed , 2121 AprApril 2026

[29] European Centre for Disease Prevention and Control and World Health Organization. 2023. Antimicrobial resistance surveillance in Europe 2023 - 2021 data. European Centre for Disease Prevention and Control, Stockholm.

[30] Amore G, Beloeil P, Fierro RG, Rizzi V, Stoicescu A, European Food Safety Authority (EFSA). 2025. Manual for reporting 2024 antimicrobial resistance data under Directive 2003/99/EC and Commission Implementing Decision (EU) 2020/1729. EFS3 22. doi:10.2903/sp.efsa.2025.EN-9238

[31] MeteoSwiss: Documentation of MeteoSwiss GridData Products. 2021. Daily precipitation (final analysis): RhiresD. Available from: https://www.meteoswiss.admin.ch/dam/jcr:4f51f0f1-0fe3-48b5-9de0-15666327e63c/ProdDoc_RhiresD.pdf

[32] MeteoSwiss: Documentation of MeteoSwiss Grid-Data Products. 2021. Daily mean, minimum and maximum temperature: TabsD, TminD, TmaxD. Available from: https://www.meteoswiss.admin.ch/dam/jcr:818a4d17-cb0c-4e8b-92c6-1a1bdf5348b7/ProdDoc_TabsD.pdf

[33] Frei C. 2014. Interpolation of temperature in a mountainous region using nonlinear profiles and non‐Euclidean distances. Int J Climatol 34:1585–1605. doi:10.1002/joc.3786

[34] MeteoSwiss. 2020. Ground-based spatial climate data – precipitation, temperature, sunshine. Available from: https://opendatadocs.meteoswiss.ch/c-climate-data/c3-ground-based-climate-data

[35] Breiman L. 2001. Random forests. Mach Learn 45:5–32. doi:10.1023/A:1010933404324

